# The effect of respiratory muscle training on children and adolescents with cystic fibrosis: a systematic review and meta-analysis

**DOI:** 10.1186/s12887-024-04726-x

**Published:** 2024-04-15

**Authors:** WenQian Cai, Meng Li, Yi Xu, Mei Li, JiaNan Wang, YaHui Zuo, JinJin Cao

**Affiliations:** 1 School of Pediatrics, Nanjing Medical University, Jiangsu Province, China; 2 Department of Nursing, Children’s Hospital of Nanjing Medical University, Nanjing, China; 3 Department of Rehabilitation Medicine, Children’s Hospital of Nanjing Medical University, Nanjing, China; 4 Department of Gastroenterology, Children’s Hospital of Nanjing Medical University, Nanjing, China

**Keywords:** Respiratory muscle training, Pediatrics, Adolescent, Cystic fibrosis, Pulmonary rehabilitation

## Abstract

**Background:**

Cystic fibrosis is a chronic genetic disease that can affect the function of the respiratory system. Previous reviews of the effects of respiratory muscle training in people with cystic fibrosis are uncertain and do not consider the effect of age on disease progression. This systematic review aims to determine the effectiveness of respiratory muscle training in the clinical outcomes of children and adolescents with cystic fibrosis.

**Methods:**

Up to July 2023, electronic databases and clinical trial registries were searched. Controlled clinical trials comparing respiratory muscle training with sham intervention or no intervention in children and adolescents with cystic fibrosis. The primary outcomes were respiratory muscle strength, respiratory muscle endurance, lung function, and cough. Secondary outcomes included exercise capacity, quality of life and adverse events. Two review authors independently extracted data and assessed study quality using the Cochrane Risk of Bias Tool 2. The certainty of the evidence was assessed according to the GRADE approach. Meta-analyses where possible; otherwise, take a qualitative approach.

**Results:**

Six studies with a total of 151 participants met the inclusion criteria for this review. Two of the six included studies were published in abstract form only, limiting the available information. Four studies were parallel studies and two were cross-over designs. There were significant differences in the methods and quality of the methodology included in the studies. The pooled data showed no difference in respiratory muscle strength, lung function, and exercise capacity between the treatment and control groups. However, subgroup analyses suggest that inspiratory muscle training is beneficial in increasing maximal inspiratory pressure, and qualitative analyses suggest that respiratory muscle training may benefit respiratory muscle endurance without any adverse effects.

**Conclusions:**

This systematic review and meta-analysis indicate that although the level of evidence indicating the benefits of respiratory muscle training is low, its clinical significance suggests that we further study the methodological quality to determine the effectiveness of training.

**Trial registration:**

The protocol for this review was recorded in the International Prospective Register of Systematic Reviews (PROSPERO) under registration number CRD42023441829.

**Supplementary Information:**

The online version contains supplementary material available at 10.1186/s12887-024-04726-x.

## Background

### Introduction

Cystic fibrosis (CF) is a multisystem autosomal recessive disease that affects approximately 90,000 individuals, according to data from CF registries worldwide [1, 2]. This is caused by mutations in the cystic fibrosis transmembrane regulator (CFTR) gene, leading to a decrease or loss in the function of the CFTR protein [2, 3]. Many studies have shown that this foundational flaw causes irreversible, progressive lung disease to start very early in life in people with cystic fibrosis (CF) [2, 4, 5]. CFTR is responsible for transporting chloride ions across the apical membrane of tissue epithelial cells, secreting bicarbonate to regulate the pH of the fluid on the airway surface, and inhibiting epithelial sodium channels (ENaC). Mutations in the CFTR gene lead to dehydration and the production of thick secretions in organs such as the reproductive, digestive, and respiratory tracts [1, 2, 6]. Thickened mucus in the lungs sticks to the surface of the airways, reducing the amount of mucociliated tracts cleared and raising the risk of infection and inflammation, which progressively damages the lungs [2, 7]. As a result, breathing becomes more difficult, and gas exchange is reduced. This lowers exercise tolerance and eventually leads to respiratory failure, the main cause of death from cystic fibrosis [2, 8].

More recently, the introduction of CFTR modulator medications can correct basic deficiencies [2, 9, 10], which in time may alter the manifestations and complications of CF. Nevertheless, a cure for this condition is not currently available, and ongoing rehabilitation is necessary due to its chronic nature. Therefore, it is crucial to develop or improve the therapeutic approaches aimed at preserving or improving lung function for the well-being of patients with cystic fibrosis. The effective intervention recently is physical exercise [2, 11], including respiratory muscle training (RMT) [2, 12]. The goal of respiratory muscle training is to enhance expiratory and/or inspiratory muscular strength and endurance in order to improve respiratory function. Respiratory muscle training has demonstrated efficacy in individuals diagnosed with chronic obstructive pulmonary disease (COPD) [2, 13, 14] and those suffering from various respiratory conditions [2, 15, 16]. It is yet unknown, nevertheless, if respiratory muscle training helps patients with cystic fibrosis. The last systematic review on this topic was published in 2020. Ten randomized controlled trials assessing the impact of respiratory muscle training on individuals with cystic fibrosis were included in the analysis. Nonetheless, the authors conclude that there is insufficient data to support the use of RMT in cystic fibrosis. The primary cause of this result is the poor methodological quality of the individual research [2].

Several research studies have suggested that respiratory muscle training could potentially improve mucus removal from the lungs, which is a fundamental aspect of preventing pulmonary infections [2, 17, 18]. At the same time, it has been proposed that respiratory muscle training could enhance lung function, exercise capacity, and health-related quality of life in patients with cystic fibrosis [19]. The aforementioned trials have a limited sample size and significant protocol variances, and despite the possible advantages of respiratory muscle training, none of them have shown strong proof of a significant increase in clinical outcomes to yet. Concurrently, recent Cochrane reviews did not consider the distinction between pediatric and adult populations with cystic fibrosis [2]. The interplay of age and disease progression in CF may lead to age-related physiological variations that can impact the adaptability and reaction of respiratory muscle training. These variations are likely to influence the effectiveness of any intervention strategies.

Thus, the primary goals of this study were to examine the efficacy of respiratory muscle training in terms of respiratory muscle function, lung function,exercise capacity, and quality of life in children and adolescents with cystic fibrosis through a systematic review and meta-analysis.

## Methods

The International Prospective Register of Systematic Reviews (PROSPERO) has the protocol for this review registered under registration number CRD42023441829. The presentation of the results of this review followed the guidelines of Preferred Reporting Items for Systematic Reviews and Meta-Analyses (PRISMA) [20].

### Criteria for eligibility

#### Study designs

All studies retrieved through the search were evaluated for eligibility based on four inclusion criteria: study or design type, population, intervention, and reported outcomes. The inclusion criteria encompassed parallel or cross-over randomized controlled trials (RCTs) comparing respiratory muscle training (RMT) with control groups.

#### Participants

Based on the World Health Organization's (WHO) classification, this meta-analysis concentrated on children (≥ 5 years old) and adolescents with CF (≤ 19 years old). They were diagnosed with CF through sweat testing, genotyping, or both. Studies involving mixed age groups of children and adults with CF were excluded from the analysis unless their data could be segregated and reported separately.

#### Interventions

Regarding interventions, the review included research that implemented a respiratory muscle training program, specifically either inspiratory muscle training(IMT) or expiratory muscle training(EMT), irrespective of the specific equipment utilized. The inclusion criteria did not impose limitations on the dosage, timing, location, or supervision of the intervention. Additionally, the review did not restrict the type of control group, whether passive (no intervention) or active (sham). However, research that combined respiratory muscle training with any other type of physical exercise training were not included in the review.

#### Outcomes

In the main included papers, it is necessary for at least one of the specified outcomes to be reported. The primary outcomes of the review focused on: respiratory muscle function such as respiratory muscle strength (maximum inspiratory pressure (MIP) and maximum expiratory pressure (MEP)) and respiratory muscle endurance, and lung function (forced expiratory volume in 1 s (FEV_1_), forced vital capacity (FVC), and cough level), where cough level assessment included quantification of forced expiratory maneuvers (peak expiratory flow (PEF)) or maximum expiratory flow achieved during cough maneuvers (peak cough flow (PCF)). Secondary outcomes include assessments of exercise capacity, quality of life, and adverse events, regardless of measurement procedures.

### Sources of information and search methodology

Until July 2023, the electronic databases that were referenced include PubMed, Web of Science, Cochrane Library, CINAHL, CNKI database, VIP database, Wan Fang database, and Chinese Biomedicine Literature Database (CBM). Depending on the database used, the search terms employed included MESH and Text words, in conjunction with free keywords utilizing the Boolean "and" and "OR" operators (Supplementary material Table S1). Furthermore, an examination was conducted on two clinical trial registries, namely the International Clinical Trials Registry Platform (ICTRP) and ClinicalTrials.gov. The reference lists of the incorporated studies and previously published systematic reviews were manually scrutinized. Only publications written in English or Chinese will be included in the literature search. Full-text versions were considered only when studies were accessible in full-text format or as conference abstracts.

### Study records

#### Selection process

The database was searched by principal investigators (CWQ). To ascertain which search results were eligible for inclusion, two reviewers (CWQ, LM) independently examined the results, and their conclusions were compared. When appropriate, we will contact the study authors for further information to address eligibility-related queries. If we are unable to come to an understanding, we shall address these matters and, if required, enlist the help of a third-party examiner (CJJ) to settle these disputes.

#### Data collection process

Two independent reviewers (CWQ, LM) extracted data using pre-structured forms to gather study characteristics and general information. We shall perform calibration activities prior to the evaluation in order to guarantee uniformity among reviewers. When a study has many publications, all reports are combined, and the data that is most complete is chosen for analysis. In addition, further information was requested from the study authors when needed. Ultimately, a third assessor (CJJ) or consensus are used to settle disagreements.

### Data items

The following details were extracted: study information (authors, publication date); sample characteristics (size, age, and FEV1); interventions (type of respiratory muscle training device, resistance settings, duration, and frequency); control groups (no treatment, sham RMT/standard care); assessment procedures, and end results.

### Risk of bias in individual studies

Two reviewers (CWQ, LM) independently evaluated the methodological rigor of the included studies using the Cochrane risk of bias tool 2 (RoB 2) [21] The methodological criteria were: (1) randomization process; (2) deviations from intended interventions; (3) missing outcome data; (4) measurement of the outcome; (5) selection of the reported results, and (6) any other identified sources of bias. Based on this tool, the studies were classified as high-risk, low-risk, or unclear. Any discrepancies were resolved through consensus. We will generate visual representations of potential bias within and across studies using RevMan 5.4 (Review Manager 5.4).

### Data synthesis

A table qualitatively described the features of the included studies. Statistical software RevMan 5.4 will be utilized to combine and calculate each outcome, adhering to the statistical guidelines outlined in the current edition of the Cochrane Handbook for Systematic Reviews of Interventions. In cases where essential data were absent from a study, corresponding authors were approached for clarification. Results were narratively described in instances where data were insufficient for meta-analysis.

#### Measures of treatment effect

When data for continuous outcomes (pulmonary function, exercise capacity and respiratory muscle function) were available, we calculated the mean differences (MD) by using pre- and post-intervention data and presented the results with 95% confidence intervals (CIs). There is currently no available data suitable for analysis of dichotomous outcomes. When aggregating findings from crossover studies for meta-analysis, we would employ the inverse variance method as suggested by Elbourne [22]. In cases where data are scarce, our approach would involve utilizing solely first-arm data or treating crossover trials as parallel trials, with the assumption that zero correlation represents the most cautious estimate.

If the study used the same tool to measure outcomes, the mean difference (MD) was utilized as the effect size. In cases where different measurement tools were utilized across studies, standard mean differences (SMDs) were employed as the effect size. All effect sizes were reported with their corresponding 95% confidence intervals (CI). Given the limited number of studies included, a random-effects model was employed in all analyses to ascertain the overall effect size, irrespective of the level of heterogeneity. Statistical significance was defined as *P* < 0.05.

#### Assessment of heterogeneity

We intended to use a standard Chi-square test with an alpha threshold of significance set at *P* < 0.05 to investigate heterogeneity between comparable studies. We would have used the I^2^ statistic to calculate the levels of heterogeneity; an I^2^ of more than 50% would be regarded as significant heterogeneity.

#### Subgroup analysis or Sensitivity analysis

The following factors were subjected to a subgroup analysis: type of respiratory muscle training.

We did not conduct a scheduled sensitivity analysis to assess the potential impact of bias in the included studies on the reliability of our findings, as there were an insufficient number of studies available for analysis.

### Confidence in cumulative evidence

Using the GRADE method, two reviewers (CWQ, LM) evaluated the quality of the evidence for each outcome [23]. The domains of bias risk, consistency, directness, precision, and reporting bias are taken into account by this paradigm. We reduced the credibility of the data by one level in cases of serious risk and by two levels in instances of very serious risk. Any discrepancies were resolved through mutual agreement.

## Results

### Search results

After conducting an electronic search, 394 records were discovered, and 19 additional records were retrieved from alternative search sources. 290 records were screened after duplicates were removed, and 239 of those were disqualified during the title and abstract review phase because they did not satisfy a minimum of one qualifying criterion. Twenty of the 51 records that were examined could not be retrieved in their entirety, and 21 were excluded (see Supplementary material Table S2 for the reasons for exclusion). The two key reasons for rejection were study population and design non-compliance. Ultimately, 10 records—representing 6 different studies—met the eligibility requirements [24–29]. Additionally, 1 ongoing study was identified (Supplementary material Table S2), which is found in ICTRP.

**Fig. 1:**
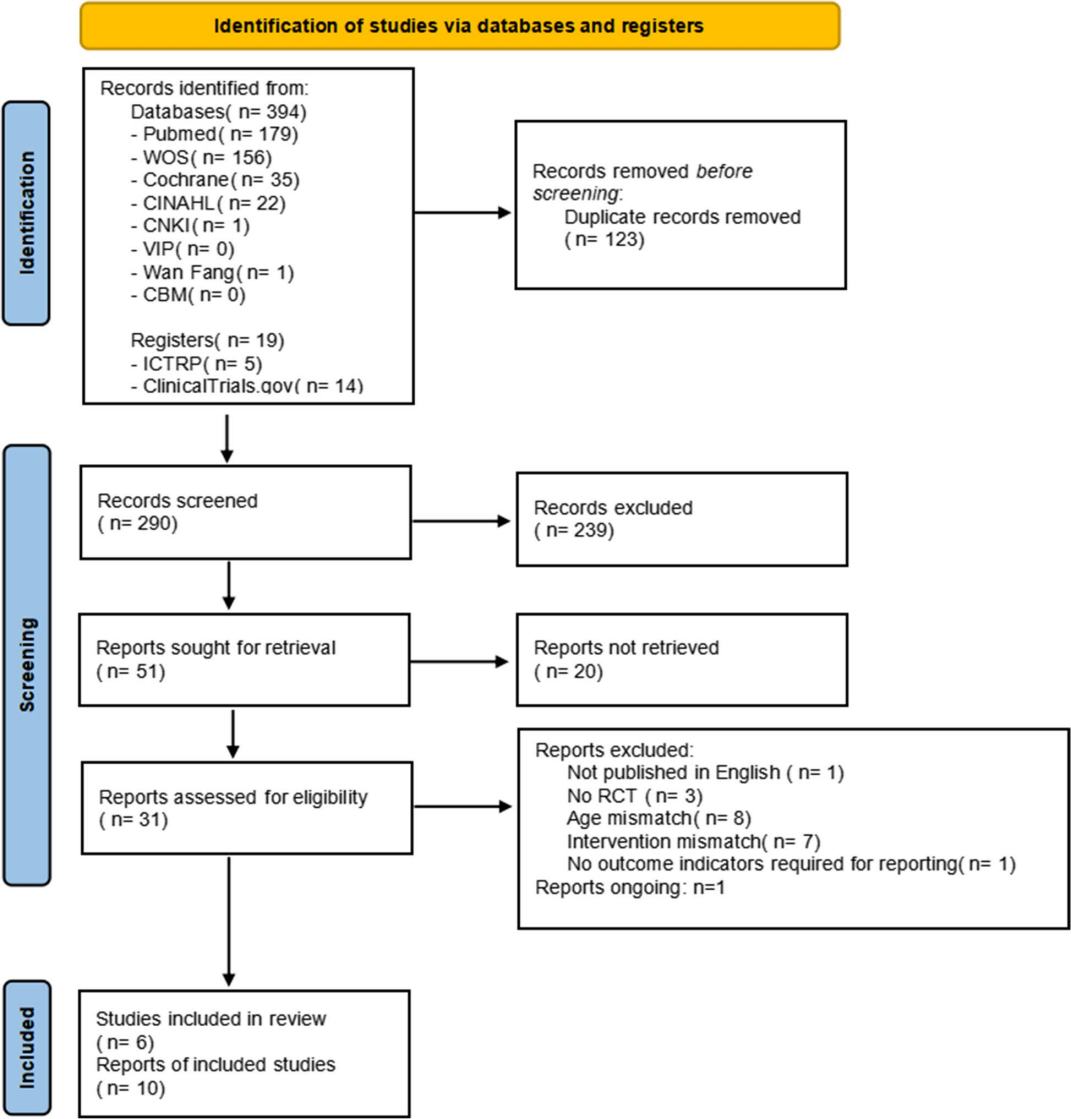
Flow diagram of the included studies

Regretfully, the reviewers were unable to get any information from the researchers. After the procedure was completed, two studies did not provide data on mean and standard deviation, leaving four studies for quantitative analysis [25–27, 29]. Figure 1 displays the PRISMA flowchart for the review procedure.

### Characteristics of the included studies

Table 1 describes the features of the six included studies. Four of the studies that met the eligibility criteria were available as full-text publications [25–27, 29], with one of them utilizing a crossover design [29]. The remaining two studies were exclusively released as conference proceedings, and all were designed as randomized controlled trials (RCTs) [24, 28]. The six studies were carried out in a variety of high-income nations. Two studies were conducted in Turkey [25, 26], two in Switzerland [28, 29], the rest in Austria [24] and the USA [27]. There were 151 total participants in the trials that were included, with a mean age of 6 to 18 years old and 50.9% of them being female.

**Table 1:** Characteristics of included studies

ID	Design	Participants		Interventions	Outcomes reported
		Experimental	Control		
Albinni 2004 [24]	RCT (conference proceedings)	*n* = 16 (gender not specified) Age range: 6–18 FEV_1_ > 30% pred	*n* = 11 (gender not specified) Age range: 6–18 FEV_1_ > 30% pred	Experimental: IMT(Respifit 1000) and CET Resistance = NR Dosage = daily × 12 week Setting = NR Progression = NR Supervision = NR Control: CET	Lung function = FEV_1_ ↔ ,FVC ↔ ,MEF 50%VC↑, MEF 25%VC↑ Inspiratory muscle strength = Pimax↑ Inspiratory muscle endurance = maximal sustained ventilatory capacity↑ Maximal exercise capacity = Vo_2_ max↑ Timing = 0, 12 wk PS: reduction of antibiotic use, markedly improved expectoration and reduced sensation of breathlessness
Emirza 2021 [25] (NCT03873688)	RCT	*n* = 15 allocated, 14 analysed (64.3% female) Age (year):12.97 (2.76) FEV_1_: 79.64 ± 27.69% pred	*n* = 15 allocated, 14 analysed (50% female) Age (year):12.13 (3.44) FEV_1_: 82.00 ± 22.49% pred	Experimental: RMT(respiratory muscle exercise device threshold PEP) and routine therapy and rehabilitation Resistance = 30% of MEP ( 5-20cmH2O) Dosage = 10 min × 2/day × 5/wk × 6 wk Setting = NR Progression = 2wkly MIP, MEP Supervision = check daily exercise records Control: shame RMT ( 5 cmH2O) + routine therapy and rehabilitation	PCF↑ Respiratory muscle strength = MIP↑, MEP↑ Lung function ↔ = FEV_1_, FVC, FEV1/FVC Exercise capacity = 6MWD↑ QoL = The Turkish version of the CFQ-R (vitality, treatment, and digestive domains of QoL in the parent↑ + physical and health domains ↑) Global rating of change score (GRoC): All patients in the groups assessed their changes as unchanged or better Timing = 0, 6 wk (The GRoC was assessed after the training was finished) PS: In comparing the two groups, changes in PCF and MEP were significantly higher in the training group. During the training, no adverse effect related to the study was experienced
Zeren 2019 [26] (NCT03375684)	RCT	*n* = 18( 50% female) Age (year):11.66 (2.42) Baseline of FEV_1_ > 70% of the predicted value FEV_1_: 79.36 ± 13.67% pred	*n* = 18( 56% female) Age (year):10.47 (2.03) Baseline of FEV_1_ > 70% of the predicted value FEV_1_: 78.69 ± 15.91% pred	Experimental: IMT( Threshold Inspiratory Muscle Trainer) + PT ( rest for at least 1 h between training) Resistance = 30% of MIP Dosage = 15 min × 2/day × 8 wk Setting = hospital, home Progression = weekly MIP Supervision = check daily exercise records Control: PT	Postural stability = BBS( static postural stability = PST; dynamic postural stability = LOST↑) Lung function = FVC↑, FEV_1_↑, PEF↑( expressed as percentages of the predicted values) MIP↑, MEP↑ Exercise capacity = 6MWD↑ PS: 14 patients in PT + IMT group (78%) and 15 patients in PT group (83%) completed all training sessions as planned. Adherence to the training program averaged 97.9% ± 4.2% in PT + IMT group and 97.5% ± 5.7% in PT group. In comparing the two groups, changes in MIP were significantly higher in the training group. No adverse effects were reported during the program
Sawyer 1993 [27]	RCT	*n* = 10( 40% female) Age (year): 11.46(2.45) FEV_1_: 89 ± 20% pred	*n* = 10( 50% female) Age (year): 9.76 (2.57) FEV_1_: 92 ± 29% pred	Experimental: IMT(threshold loading device) Resistance = 60% Plmax ( Starting from -7cmH_2_O) Dosage = 30 min/ day x 7 days/ wk x 10wk Setting = home Progression = weekly MIP Supervision = 3 home visits per week by a nurse + diary Control: 10% Pimax	Inspiratory muscle strength = Pimax↑ Maximal exercise testing = Exercise time on the treadmill↑ Lung function = Improvement in lung function outcomes required in systematic reviews was not reported
Bieli 2014 [28]	RCT (conference proceedings)	*n* = 16 (gender not specified) Age range: 6–18 FEV_1_: 87.0 ± 25.8% pred		A Group: RMET( SpiroTiger®) x 8wk + chest physiotherapy x 8wk B Group: chest physiotherapy x 8wk + RMET x 8wk	Respiratory endurance↑ Exercise endurance↑ QoL ↔ Lung function ↔ Clinical score ↔
Bieli 2017 [29]	RCT(two-sequence, two-period crossover design)	intervention/control( IC): *n* = 11( 54.5% female) Age (year): 15.4( 12.0; 16.6) FEV_1_ > 40% pred	control/intervention( CI): *n* = 11( 54.5% female) Age (year): 13.2( 11.9; 17.8) FEV_1_ > 40% pred	Intervention period: RMET by voluntary eucapnic hyperventilation( SpiroTiger®) Resistance = The initial training conditions were defined by breathing performance representing 50% MVV Dosage = 5–10 min × 2/day × 5/wk x 8wk ( two periods separated by a 1-week washout) Setting = home Progression = NR Supervision = supervised by a physiotherapist weekly Control period: standard chest physiotherapy	RME test: time to exhaustion↑ Exercise testing ↔ : time to exhaustion( perform on a cycle ergometer in the sitting position) Lung function( expressed as z-scores) ↔ : FEV_1_, FVC, MEF 75/25 QoL: CFQ ↔ CFCS ↔ Timing = 0, 9, 17wk

(1) Age is expressed in all studies as mean ± standard deviation or range; (2) ↔ not statistically signifificant changes; ↑statistically signifificant improvement

*Abbreviations*: *RCT* randomized clinical trial, *FEV*_*1*_ forced expiratory volume in one second, *IMT* inspiratory muscle training, *CET* cycle ergometer training, *MEF 50%VC* mean expiratory flow at 50% of FVC, *MIP (Pimax)* maximal inspiratory pressure, *Vo*_*2*_*max* maximal oxygen uptake, *RMT* respiratory muscle training, *PEP* positive expiratory pressure, *MEP* maximal expiratory pressure, *PCF* peak cough flow, *FVC* forced vital capacity, *6MWD* 6-min walking distance, *CFQ-R* a revised version of the Cystic Fibrosis Questionnaire, *QoL* quality of life, *GRoC* Global rating of change score, *PT* physical therapy, *BBS* Biodex Balance System, *PST* Postural Stability Test, *LOST* Limits of Stability Test, *PEF* peak expiratory flow, *RMET* respiratory muscle endurance training, *MVV* maximal voluntary ventilation, *MEF 75/25* mean expiratory flow at 75–25% of FVC, *CFCS* Cystic Fibrosis clinical score, *NR* not reported

The included studies varied greatly in terms of the training level and methodology. For the intervention, three of the selected studies applied inspiratory muscle training [24, 26, 27], while one used expiratory muscle training [25]. The four aforementioned studies focused on respiratory muscle strength training using pressure threshold loading. In contrast, Beilil et al. [28, 29] conducted respiratory muscle endurance training. The intensity of training varied across the studies, with most targeting a range of 30% to 60% of maximum inspiratory pressure and/or maximum expiratory pressure. The progression was based on a periodic reassessment of the maximum inspiratory/expiratory pressure. RMT was conducted for a duration of 10–30 min, once or twice a day, 5–7 days per week, over a total period of 6 to 12 weeks. In the control groups, two studies used sham respiratory muscle training, using 5 cmH_2_O of load or 10% of the maximum inspiratory pressure [25, 27], and three studies used standard care as the control [26, 28, 29]. Additionally, Albinni et al. [24] conducted a comparison between the use of a cycle ergometer alone and the addition of inspiratory muscle training.

Also, there was a wide range in the outcome measures that the research chose. All studies reported at least one measure of lung function, principally FEV_1_ and FVC. Expiratory muscle training by Emirza et al. also reported PEF. Exercise capacity was reported by all studies, specifically 6-min walking distance (6MWD) [25, 26], maximum oxygen uptake (VO_2_max) [24]and exercise duration [27, 28, 29]. Three studies used the Cystic Fibrosis Clinical Score (CFCS) or the Cystic Fibrosis Questionnaire (CFQ) to measure health-related quality of life [25, 28, 29]. Two studies assessed the level of adherence with the training regimen [25, 26] 97.9% (SD 4.2) and 97.5% (SD 5.7) for the experimental group and 97.5% (SD 5.7) for the control group, respectively, were the findings of one study; [26] Another study reported excellent adherence without providing specific numerical data [25].

### Risk of bias assessment

**Fig. 2:**
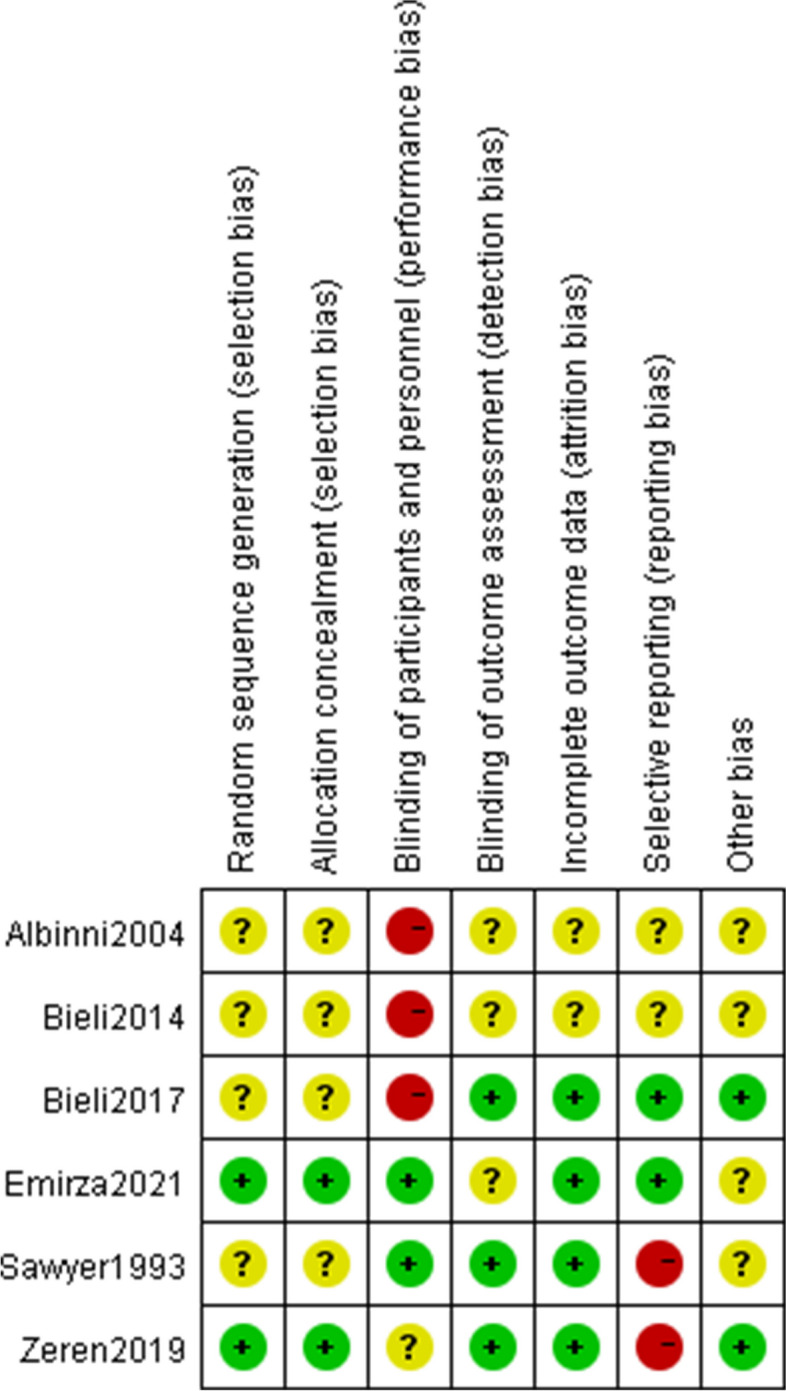
Risk of bias summary

The majority of included studies had bias risk across all ROB2 domains. The evaluation results were shown in Fig. 2.

Four of the studies that were considered had ambiguous allocation concealment and random sequence creation (randomness of participant allocation) [24, 27–29]. Most just mentioned that the participants were placed into their groups at random, they did not elaborate on the randomization procedure. It is quite challenging to conduct double blind research in respiratory muscle training. Two studies were rated as having low performance bias because of the use of sham controls [25, 27]. In the study of zeren et al., although patients knew which interventions they were doing, the control group also measured maximum inspiratory pressure values weekly to mitigate the impact of the interventions, so the risk of bias of the zeren et al. study was unclear [26]. Three studies presented with low risk of detection bias (blinding to the outcomes), the assessors did not know the allocation scheme [26, 27, 29], whilst the other studies were unclear due to lack of information.

Regarding incomplete outcome data (attrition bias), the risk of two conference proceedings was unclear [24, 28] and the remaining studies were at low risk. The intention-to-treat concept was addressed in the Bieli et al. study. Of the 22 participants, 6 withdrew from the trial, and 4 of them stopped during the control period, indicating that the withdrawal was not directly related to the intervention. Moreover, participants who withdrew did have a tendency to age and have features of more advanced lung disease. We assessed the Bieli et al. study as having a minimal risk of bias [29]. Additionally, we evaluated another study as having a low risk of bias, although some participants did not complete all training sessions, the reported good adherence was insufficient to affect the outcome analysis [26]. Data were dropped in two studies, both explaining the reasons for the dropout and did not affect the outcome analysis [25, 27].

For reporting bias/selective reporting (selection of outcomes reported in published articles), two studies were classified as high risk because they did not report all prespecified outcomes [26, 27]. Two studies provided data on all chosen outcome measures, thus indicating a low likelihood of selective reporting bias in the studies [25, 29]. The publications did not provide enough information to assess the risk of bias, and as a result, they have been deemed to have an unclear risk of bias [24, 28].

### Effects of intervention and certainty of evidence

**Fig. 3:**
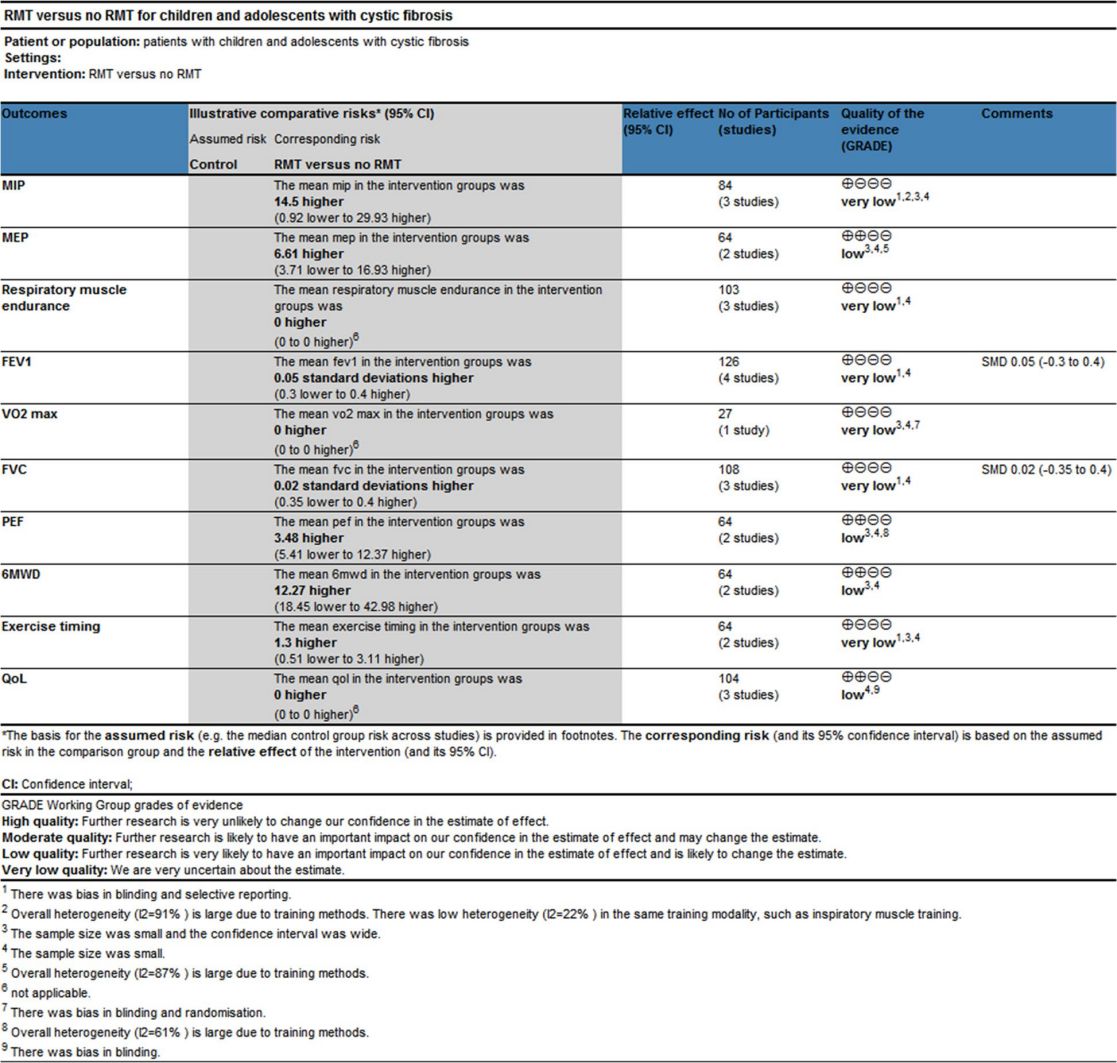
GRADE Summary of findings

The quantitative analysis did not include two of the papers that were part of this review [24, 28]. One study provided information on the overall research participants without specifying for each intervention and control group [28]. It is impossible to estimate the standard deviation from another study because the exact *p*-value and 95% confidence interval of the mean difference within or between groups were not provided [24]. As a result, meta-analyses based on the final four studies were carried out. For every meta-analysis, the quality of the evidence was graded as poor or very low (Fig. 3), mostly because of the imprecision resulting from the small sample size overall and the quality of the studies.

#### Comparison 1 respiratory muscle strength and endurance

**Fig. 4:**
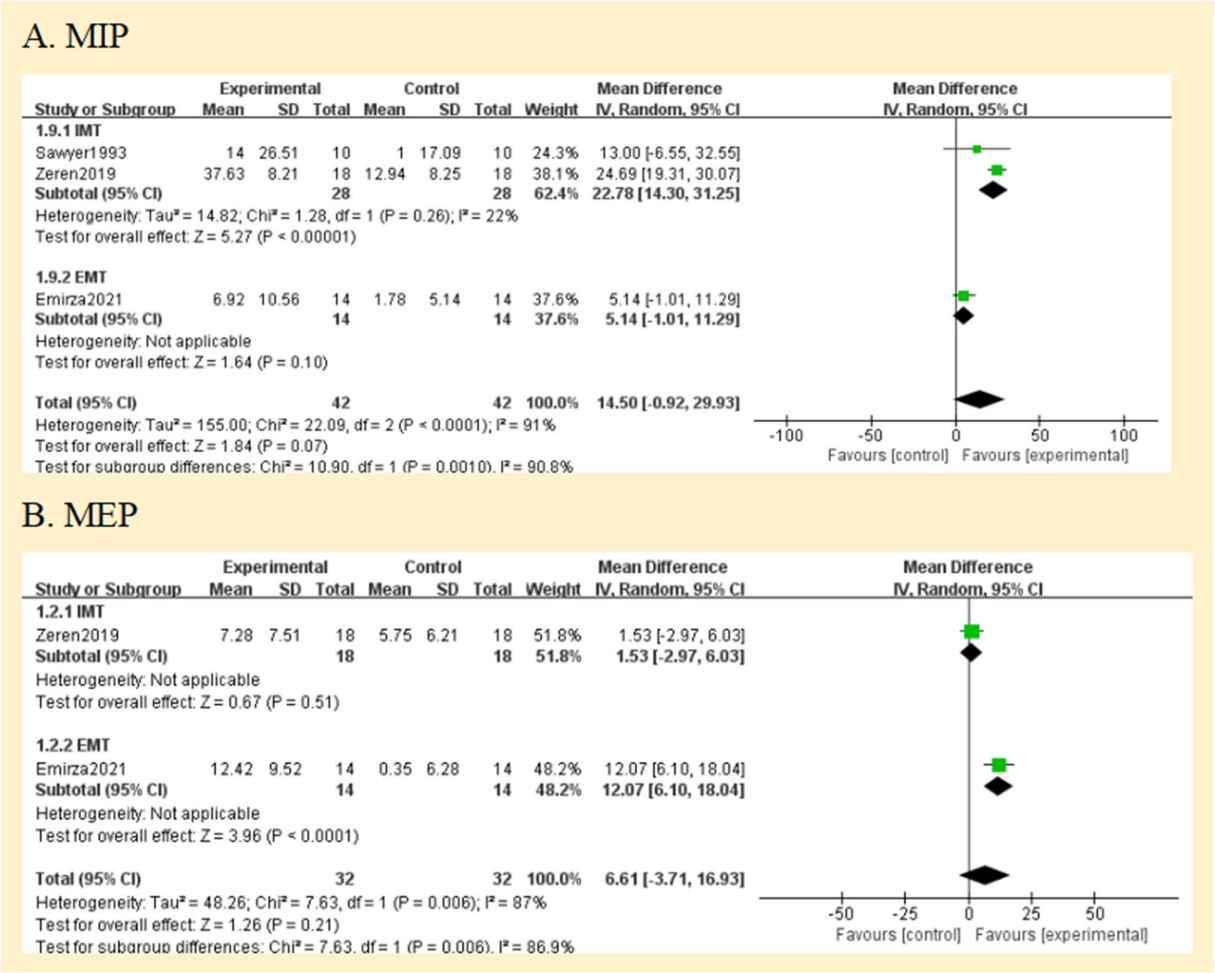
Pooled analysis of respiratory muscle strength. Abbreviation: IMT, inspiratory muscle training; EMT, expiratory muscle training; MIP, maximal inspiratory pressure; MEP, maximal expiratory pressure

Four studies had documented maximum inspiratory pressures [24–27]. Comparisons between the RMT and control groups were presented based on pooled data analysis of three studies (84 patients) [25–27]. The overall MD was 14.50 cmH_2_O [95%CI -0.92; 29.93] and overall effect Z = 1.84 (*p* = 0.07) (Fig. 4A) (very low certainty of evidence; see Fig. 3). One study was excluded from the analysis due to insufficient data [24]. The omitted study identified a significant maximal inspiratory pressure improvement in the experimental group. Subgroup analyses of studies using IMT alone revealed higher improvements in the experimental groups' maximal inspiratory pressure when compared to the control groups; The overall MD was 22.78 cmH_2_O [95%CI 14.30; 31.25]. The heterogeneity of the comparison was low (I^2^ = 22%) (Fig. 4A). Nevertheless, no significant differences were seen in subgroup analyses conducted just utilizing EMT.

The maximal expiratory pressure (MEP) was assessed in 2 studies [25, 26]. MEP also did not favour experimental interventions overall. The overall MD was 6.61 cmH_2_O [95%CI -3.71; 16.93] (Fig. 4B) (low certainty of evidence; see Fig. 3). But in the EMT subgroup, maximal expiratory pressure favour experimental interventions [MD = 12.07 cmH_2_O, 95% CI 6.10; 18.04].

The endurance of respiratory muscles was evaluated in 3 studies [24, 28, 29] using varying methodologies, two studies had insufficient data [24, 28], so a meta-analysis was not possible. The quality of evidence was deemed to be of very low (Fig. 3). According to two studies, the training group's respiratory muscle endurance improved (*P* < 0.01) [24, 28]. At a 70% MVV breathing performance, Bieli et al. [29]similarly discovered that the training group's respiratory muscle endurance was longer (*P* < 0.01).

#### Comparison 2 lung function

**Fig. 5:**
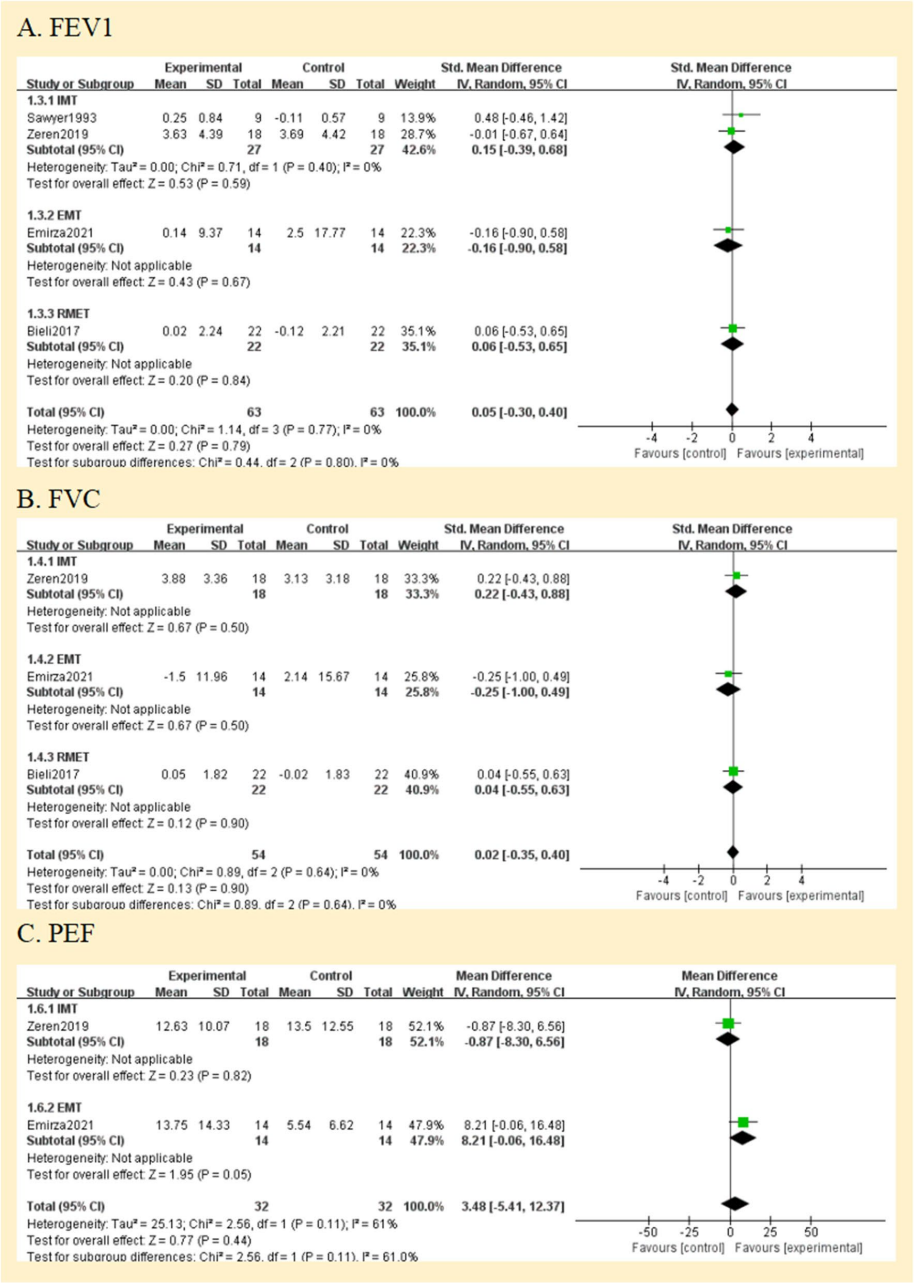
Pooled analysis of pulmonary function. Abbreviation: IMT, inspiratory muscle training; EMT, expiratory muscle training; RMET: respiratory muscle endurance training; FEV1, forced expiratory volume in 1 s; FVC, forced vital capacity; PEF: peak expiratory flow

All studies assessed lung function. They reported lung function in eitherlitres (L) [27, 28], % predicted [25, 26] or using the z score; [29] one study did not define the unit of measurement in the two published abstracts [24]. Therefore, analyses were performed with SMD. The forced expiratory volume in one second (FEV_1_), forced vital capacity (FVC), and peak expiratory flow (PEF) were compared between the RMT and control groups in Fig. 5. Regarding the assessment of the effect on FEV_1_ and FVC, there was no heterogeneity between the trials (I^2^ = 0%; *p* = 0.77 and I^2^ = 0%; *p* = 0.64, respectively); however, PEF was very significant. (I^2^ = 61%; *p* = 0.11) (Fig. 5A, B, and C). None of the pooled parameters showed any discernible variations. Two studies that were not included in the meta-analysis reported no significant benefit of RMT in terms of lung function [24, 28]. The only one is that in the subgroup analysis of PEF, EMT has significant benefits for PEF [MD = 8.21, 95% CI -0.06; 16.48]. We judged the quality of the evidence for FEV_1_ and FVC to be very low, and the quality of the evidence for PEF to be low (Fig. 3).

#### Comparison 3 exercise capacity

**Fig. 6:**
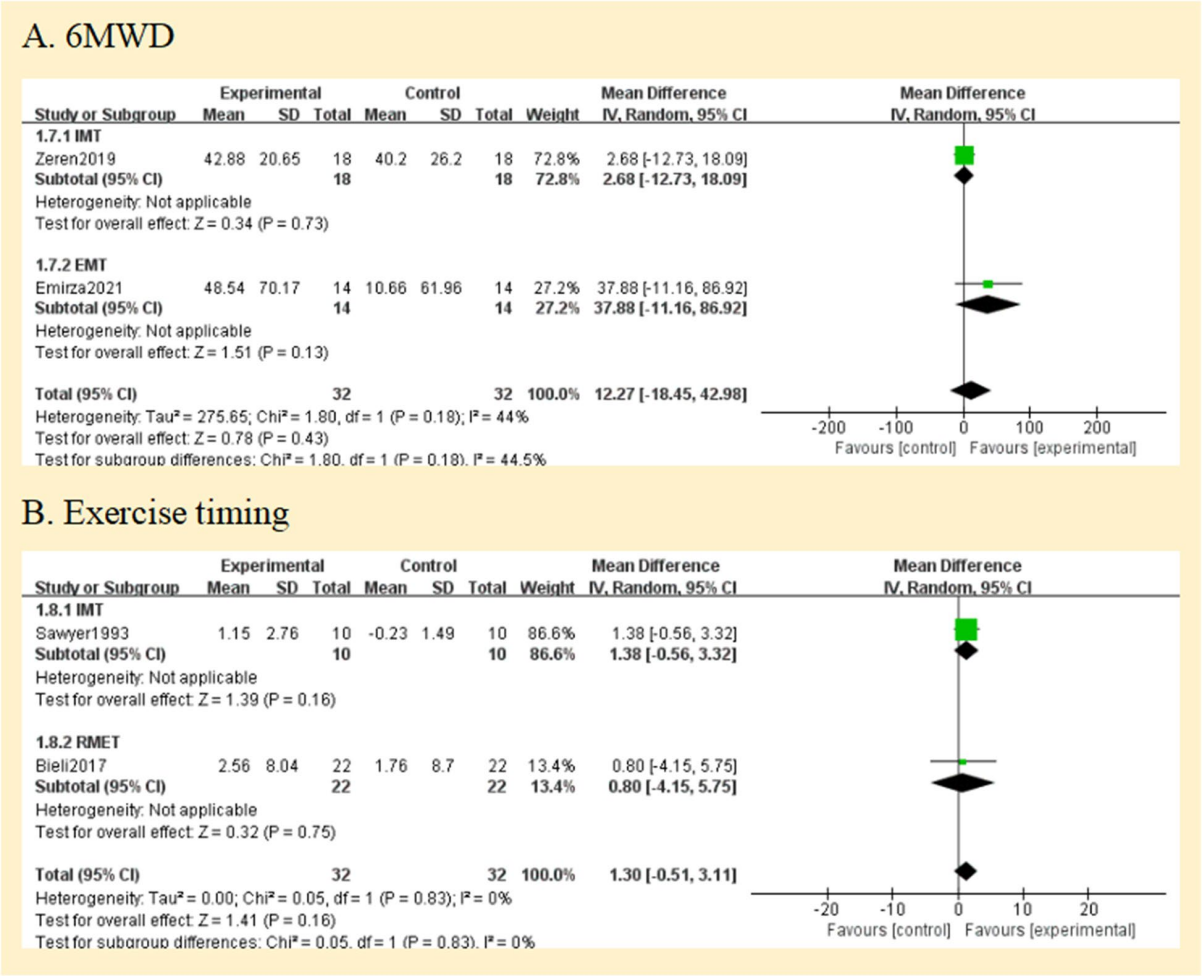
Pooled analysis of exercise capacity. Abbreviation: IMT, inspiratory muscle training; EMT, expiratory muscle training; RMET, respiratory muscle endurance training; 6MWD, 6-min walking distance

Two studies reported the exercise capacity as measured by the distance covered during the 6-min walking test [25, 26]. While the distance achieved improved from baseline in both studies during the study, there was no difference in between group comparisons of change from baseline. Pooled data analysis showed no significant differences for the distance walked[MD = 12.27 m, 95% CI -18.45; 42.98] between groups (low certainty of evidence; see Fig. 3). The level of heterogeneity was moderate (I^2^ = 44%; *p* = 0.18) (Fig. 6A).

Using the duration of the activity, three studies revealed the exercise capacity [27–29]. Analysis of pooled data from two studies (64 patients) compares the RMT and control groups [27, 29]. The overall MD was 1.30 min [95%CI -0.51; 3.11] and overall effect Z = 1.41 (*p* = 0.16) (Fig. 6B) (low certainty of evidence; see Fig. 3). A study that compared groups indicated that working at 60% of maximal effort resulted in a 10% improvement (*P* < 0.03) [27], but our subgroup analysis showed no statistically significant difference between groups (*p* = 0.16).

Regarding maximum exercise capacity, this result was documented in one study [24]. Maximal exercise capacity was defined as maximal oxygen uptake (Vo_2max_). It only reported within-group improvements, with no data to allow inclusion in our analysis. We assessed the evidence quality to be of a very low standard (Fig. 3).

#### Health-related quality of life

Three studies reported a measure of health-related quality of life by using the CFQ or CFQ Revised (CFQ-R) [25, 28, 29]. Meta-analysis was impossible due to different versions. We judged the quality of the evidence to be low (Fig. 3). Emriza et al. used a revised version of the Turkish Cystic Fibrosis Questionnaire (CFQ-R) [25]. The Turkish version of the Cystic Fibrosis Questionnaire-Revised (CFQ-R) consists of child form, parent form, and adolescent form over multiple domains(physical, emotion, vitality, school, eat, treat, social, body, health, weight, respiratory, digestive, role) [30]. For each CFQ-R domain, Emriza provided the overall score both before and after training. In the training group, there were significant changes in the children's physical function scores and parents' physical function, vitality, and health perception (*P* < 0.05). In the comparison between the two groups, parents had variations in their scores on treatment burden, digestive symptoms, and vitality (*P* < 0.05) [25]. Bieli et al. used German adaptation of the CFQ-14 + , but the study participants were not all over 14 years old, and the report did not explain this [29]. Bieli's utilization of the CFQ revealed no discernible disparity in the health-related quality of life across the various treatment groups.

The CF clinical score (CFCS), which Bieli also used to gauge symptom severity, shows overall symptom severity. However, neither at baseline nor during the intervention, there was a difference in symptom severity between the two groups [28, 29].

## Discussion

This meta-analysis summarizes and analyzes the available evidence on the effects of RMT in children and adolescents with cystic fibrosis. This review examines the impact of age on the progression of CF and is the initial review of respiratory muscle training in children and adolescents with CF. The results suggest that the RMT program is an effective intervention to improve inspiratory/expiratory muscle strength in children and adolescents with cystic fibrosis. It also indicates that it may have a positive effect on respiratory muscle endurance with no adverse effects. Expiratory muscle training alone was superior to the control group in improving PEF. RMT did not improve lung function (FEV1, FVC), and the results were inconclusive regarding the benefits of exercise capacity and HRQoL.

The review's overall high risk of bias and small sample size have also led to a low or very low quality of evidence supporting these findings. Of the 6 included studies, only four (including 108 participants) were fully published papers [25–29], highlighting the need for further research. Summary of conference proceedings limits the amount of detailed data provided, thereby limiting the data that can be extracted and reducing the rigor of the process.

Training regimens in studies varied widely, and no recommendations have been made on the load, intensity, or duration of training. Of the studies included in this review, 67% used a threshold loading device to transmit resistance (focusing on respiratory muscle strength) with a target intensity of 30% or 60% of maximum respiratory muscle strength [24–27]. This result is consistent with the manufacturer's recommendations for effective inspiratory muscle training (strong breathing), which recommends at least 30% intensity. 33% hyperventilation by autonomic eucapnia (focusing on respiratory muscular endurance) [28, 29].

Descriptive analysis of the studies showed that the indicators that best detect the effectiveness of the respiratory muscle training program were maximal inspiratory pressure, maximal expiratory pressure, and respiratory muscle endurance time. The included studies all showed improvements in respiratory muscle function in the trial group, but pooled meta-analyses of training interventions had no significant benefit. For the pre-specified subgroup analyses, due to the limited number of studies, only two studies were included in the inspiratory muscle training subgroup analyses, which showed greater improvement in maximal inspiratory pressure in the trial groups [26, 27]. Individual studies that failed to perform subgroup analyses due to limited data also showed that EMT significantly improved expiratory muscle strength [25]. In addition, IMT does not improve expiratory muscle strength, and vice versa. This is consistent with respiratory muscle training results for other chronic respiratory diseases [31, 32]. When the proper physiological load is applied, respiratory muscles respond to training in a manner comparable to that of any skeletal muscle since they are both physiologically and functionally skeletal muscles [33]. So if the patient can tolerate it, is it possible to conduct joint training to study the effect of the intervention, after all, meta-analyses have demonstrated that IMT + EMT can enhance both inspiratory and expiratory muscle strength [34].

Only three studies evaluated respiratory muscle endurance, and all of them had positive findings, although using two distinct techniques for assessment [24, 28, 29]. The results coincide with those observed in asthma [35]. Despite the fact that respiratory muscle pressure is the most widely used indicator of respiratory muscle function in clinical settings, endurance components of respiratory muscle function are important because of their impact on daily activities and their role in facilitating gas exchange and ventilation during physical activity [36]. Sawyer et al. [27] tried using an incremental loading procedure to assess the maximum working capacity of the inspiratory muscles, but many children did not perform this procedure correctly and therefore did not report results. Further assessments of respiratory muscle endurance could be beneficial in order to better comprehend the efficacy of RMT. Consequently, we think that assessing respiratory muscle endurance should be a part of future study, and it can be challenging to discover an appropriate way to measure it in youngsters.

According to the meta-analysis's findings, there was not a significant difference between the experimental and control groups' lung function tests (such as FEV_1_ and FVC). There are other possible contributing variables to this finding. Firstly, inspiratory interventions do not alter expiratory indicators. Studies employed exhalation measurements primarily because they are applicable to ordinary clinical use and illness progression monitoring. Secondly, the effects of respiratory muscle training are short-lived and not sufficient to halt the natural decline of the lungs in these patients. Lastly, the baseline lung function, such as FEV_1_ and FVC, near to the normal prediction range, and the effect of respiratory muscle training on them would not be evident given the features of the group included in the studies. This hypothesis may explain why Enright et al. achieved significant improvements in spirometry, with baseline predictive values of 64% for FEV_1_ and 53% for FVC in their study, and the severity of lung deterioration in patients may have enhanced the benefit of RMT on spirometry [19]. Future studies may involve a larger sample of individuals with reduced lung function to enhance the generalizability of the findings. Additionally, extending the follow-up period could help confirm the long-term effectiveness of respiratory muscle training in children and adolescents with cystic fibrosis.

Cough is a part of life for people with cystic fibrosis (CF), is the main mechanism of secretion clearance [37], and is directly related to respiratory muscle strength [38, 39]. Quantitative assessment of cough capacity is typically conducted by measuring PCF, but there are currently no internationally accepted guidelines for PCF testing [40]. The study by Morrow et al. [41] confirmed a significant positive linear correlation between PEF and PCF in children with neuromuscular disorders (NMDs), showing strong consistency. PEF can be used as an alternative test to assess the effectiveness of coughing. We combined PCF and PEF to discuss the effectiveness of respiratory muscle training for cough. According to reports, a technique to boost cough should be added to the treatment if the PCF is less than 270 L/min, which is the minimum required for an effective cough [42]. Two studies reported PEF or PCF, both parameters that can objectively measure cough capacity [43]. Emriza et al. [25] found that most patients with CF had a low effective cough score before training, for which expiratory muscle training was performed. The result showed a significant increase in PCF of 52.42 ± 51.91 L/min in the training group. Zeren et al. [26] used inspiratory muscle training, with PEF expressed as a percentage of predicted values, and the results showed that inspiratory muscle training did not confer significant improvement in PEF. Children and adolescents with neuromuscular disorders and chronic lung diseases (CF, bronchiectasis, postinfectious bronchiolitis obliterans) were included in Rodriguez et al. [44]. According to this study, patients' PCF of 16 L/min is improved by IMT + EMT. We think the impact on PCF is greater when expiratory muscle training is used alone.

Notably, among patients with CF, exercise capacity is a major predictor of both mortality risk and deterioration [45, 46]. The higher the level of aerobic fitness in people with cystic fibrosis, the lower the risk of death. But only one study in the review reported maximum oxygen uptake (Vo_2max_), it reported a significant improvement in Vo_2max_ in the inspiratory muscle training group, but not in the control group [24]. Therefore, in the future, it is necessary to further explore the aerobic fitness ability of patients with CF after RMT, which will be important for determining the optimal respiratory muscle training regimen.

A great number of patients with CF need lifetime care, which entails frequent admissions and a demanding daily treatment schedule. The impact of this heavy treatment load on health-related quality of life (HRQoL) is substantial [47, 48]. We must not only consider the effectiveness of interventions, but also assess how patients perceive the benefits of their treatment, which should be particularly important for patients with cystic fibrosis exhibiting chronic, long-term characteristics. However, only three of the studies included in the review reported having used an outcome measure assessing health-related quality of life [25, 28, 29]. We believe that this is a significant omission from other studies and severely limits the external validity of the research base. Bieli reported that neither CFQ nor CFCS improved [29]. In the study of Emirza et al., the physical function of patients and parents in the training group was improved, and the treatment burden of parents was reduced [25]. They also offered significant evidence regarding the relative value of interventions from the perspective of the patient and parent. In health technology assessments, quality of life scores can be utilized in conjunction with other scientific information to support financing or regulatory decisions for cystic fibrosis treatment.

## Limitations

The RMT treatment intervention for CF with children and adolescents has two limitations. First, the data were mainly from small clinical trials and were highly heterogeneous, for example, The ability to detect treatment effects was hampered by the inconsistent methodological quality of the included studies, heterogeneity in the results used in the studies, the units of measurement for some outcomes, and the methods and degrees of determining and reporting participants' clinical status. Furthermore, the trials' follow-up periods were insufficiently short (12 weeks was the longest regimen) and the effects of long-term use were not entirely evident, thus it could be worthwhile to prolong the intervention time to six months or perhaps a year.

The review is further limited by the following: Two studies were not included in the quantitative analysis for previously stated reasons. The technique used to assess for publication bias (funnel plots and statistical tests) is another factor. It is advised to include at least 10 studies for a more reliable assessment, but since the current review only used up to six studies for this assessment, this suspicion cannot be confirmed [49].

## Conclusions

### Future research

Determining the most appropriate respiratory muscle training regimen is a major challenge for respiratory muscle training studies, and further high-methodologically quality studies are necessary to distinguish the most significant advantages associated with different types of resistance loads, including pressure thresholds and flow resistance loads, and volumetric loading (i.e., autonomous hyperventilation), and importantly, to clarify the optimal training volume and intensity of the respiratory muscle training protocol, as well as to determine the range of variation in results associated with respiratory muscle training.

### Conclusion

It's still uncertain if respiratory muscle training can be a helpful therapeutic strategy in the treatment of cystic fibrosis. When determining whether to use respiratory muscle training as a form of exercise therapy for children and adolescents with cystic fibrosis, healthcare professionals are advised to consider each case individually.

### Supplementary Information

**Supplementary Material 1. **

**Supplementary Material 2. **

## Data Availability

No datasets were generated or analysed during the current study.

## Abbreviations

CF: Cystic fibrosis
CFTR: Cystic fibrosis transmembrane regulator
RMT: Respiratory muscle training
IMT: Inspiratory muscle training
EMT: Expiratory muscle training
COPD: Chronic obstructive pulmonary disease
PRISMA: Preferred Reporting Items for Systematic Reviews and Meta-Analyses
RCT: Randomised controlled trials
RoB 2: Cochrane risk of bias tool 2
MD: Mean differences
CIs: Confidence intervals
RR: Risk ratios
CET: Cycle ergometer training
FEV1: Forced expiratory volume in the first second
FVC: Forced vital capacity
MIP: Maximum inspiratory pressure
MEP: Maximum expiratory pressure
PEF: Peak expiratory flow
PCF: Peak cough flow
PEP: Positive expiratory pressure
6MWD: 6-Minute walking distance
CFQ-R: A revised version of the Cystic Fibrosis Questionnaire
GRoC: Global rating of change score
HRQoL: Health-related quality of life
CFCS: Cystic fibrosis clinical score
NMDs: Neuromuscular Disorders
