# Supplementary material for: The effect of respiratory muscle training on children and adolescents with cystic fibrosis: a systematic review and meta-analysis
Source: BMC Pediatr. 2024 Apr 15;24:252. doi: 10.1186/s12887-024-04726-x (PMC11017573; doi:10.1186/s12887-024-04726-x)
Supplement: Supplementary file 1 — Supplementary Material 1. [file 12887_2024_4726_MOESM1_ESM.docx]

**Search Strategy for Pubmed**

| #1 | "Cystic fibrosis"[Mesh] | 39,904 |
| --- | --- | --- |
| #2 | Cystic fibrosis[Title/Abstract] | 51,104 |
| #3 | #1 or #2 | 56,686 |
| #4 | (respiratory muscle training) OR (inspiratory muscle training)) OR (expiratory muscle training) | 9,181 |
| #5 | #3 and #4 | 179 |

**Search Strategy for Web of Science**

| #1 | Cystic fibrosis | 94,469 |
| --- | --- | --- |
| #2 | ((TS=(respiratory muscle training)) OR TS=(inspiratory muscle training)) OR TS=(expiratory muscle training) | 9,923 |
| #3 | #2 AND #1 | 156 |

**Search Strategy for Cochrane**

| #1 | MeSH descriptor: [Cystic Fibrosis] explode all trees | 2,520 |
| --- | --- | --- |
| #2 | ("Cystic fibrosis"):ti,ab,kw | 6,264 |
| #3 | #1 or #2 | 6,264 |
| #4 | ("Respiratory Muscle Training" or "inspiratory Muscle Training" or "expiratory muscle training"):ti,ab,kw | 1,654 |
| #5 | #3 and #4 | 35 |

**Search Strategy for CINAHL**

| #1 | (MH "Cystic Fibrosis") | 8,697 |
| --- | --- | --- |
| #2 | SU Cystic fibrosis | 4,902 |
| #3 | #1 or #2 | 4,902 |
| #4 | respiratory muscle training OR inspiratory Muscle Training OR expiratory muscle training | 1,558 |
| #5 | #3 and #4 | 22 |

**Search Strategy for CNKI database**

| SU=囊性纤维化 AND SU=（呼吸肌训练 + 通气肌肉训练 + 呼气肌训练 + 吸气肌训练） | 1 |
| --- | --- |

**Search Strategy for VIP database**

| [(题名或关键词=囊性纤维化 AND ((任意字段=呼吸肌训练 OR 任意字段=吸气肌训练) OR 任意字段=呼气肌训练))](http://qikan.cqvip.com/Qikan/search/index?LngMySearHistoryIdGuid=bf9d7a06-ce65-484b-9f70-01e950c9fd53&from=Qikan_Article_History) | 0 |
| --- | --- |

**Search Strategy for Wan Fang database**

| 主题:(囊性纤维化) and 主题:(呼吸肌训练 or 吸气肌训练 or 呼气肌训练) | 1 |
| --- | --- |

**Search Strategy for CBM**

| ("囊性纤维化"[标题:智能]) AND ("呼吸肌训练"[常用字段:智能] OR "吸气肌训练"[常用字段:智能] OR "呼气肌训练"[常用字段:智能]) | 0 |
| --- | --- |

**Search Strategy for ICTRP**

| Title | Cystic fibrosis |  |
| --- | --- | --- |
| Intervention | "Respiratory Muscle Training" or "inspiratory Muscle Training" or "expiratory muscle training" |  |
|  | Title and intervention | 5 |

**Search Strategy for ClinicalTrials.gov**

| Title | Cystic fibrosis |  |
| --- | --- | --- |
| Intervention | "Respiratory Muscle Training"  "inspiratory Muscle Training"  "expiratory muscle training" |  |
|  | Title and intervention | 14 |
