# Supplementary material for: The effect of respiratory muscle training on children and adolescents with cystic fibrosis: a systematic review and meta-analysis
Source: BMC Pediatr. 2024 Apr 15;24:252. doi: 10.1186/s12887-024-04726-x (PMC11017573; doi:10.1186/s12887-024-04726-x)
Supplement: Supplementary file 2 — Supplementary Material 2. [file 12887_2024_4726_MOESM2_ESM.docx]

**Table S2** Reasons for exclusion of studies reviewed in full text.

| ID | Title | Reason for exclusion |
| --- | --- | --- |
| **Karila2010** | Physical activity and exercise training for patients with cystic fibrosis | Not published in English |
| **Santana2014**  **( NCT01706445)** | Benefits of combining inspiratory muscle with ‘whole muscle’ training in children with cystic fibrosis: a randomized controlled trial | It is a joint training, not a single respiratory muscle training |
| **ZACH1982** | Cystic fibrosis: physical exercise versus chest physiotherapy. | Intervention mismatch，no RMT |
| **McCreery2021** | Effects of Inspiratory Muscle Training on Heart Rate Variability in Children with Cystic Fibrosis: A Pilot Study | Outcome - did not report outcomes included in this review |
| **Reix2012** | Exercise with incorporated expiratory manoeuvres was as effective as breathing techniques for airway clearance in children with cystic fibrosis: a randomised crossover trial | Intervention mismatch，no RMT |
| **Zanchet2006** | Influence of the technique of re-educating thoracic and abdominal muscles on respiratory muscle strength in patients with cystic fibrosis | Just health education about RMT |
| **Gruber2014** | Interval exercise training in cystic fibrosis — Effects on exercise capacity in severely affected adults | Age mismatch  Intervention mismatch，no RMT |
| **Santana2012** | Intrahospital Weight and Aerobic Training in Children with Cystic Fibrosis: A Randomized Controlled Trial | Intervention mismatch，no RMT |
| **Gambazza2014** | Isocapnic hyperpnea with a portable device in Cystic Fibrosis: an agreement study between two different set-up modalities | Age mismatch |
| **Cahalin2015** | Novel Methods of Inspiratory Muscle Training via the Test of Incremental Respiratory Endurance (TIRE) | No RCT |
| **Prasad2000** | Physiotherapy in cystic fibrosis | No RCT |
| **Mellins1974** | Pulmonary physiotherapy in the pediatric age group | No RCT |
| **Sartori2008** | Respiratory training with a specific device in cystic fibrosis: A prospective study | Age mismatch |
| **Holzer1984** | The effect of a with home exercise programme in children  cystic fibrosis and asthma | Intervention mismatch，no RMT |
| **Delk1993** | The effects of biofeedback assisted breathing retraining on lung functions in patients with cystic fibrosis | Age mismatch;  Only the use of an incentive inspirometer is reported, and it is uncertain whether it is the respiratory muscle training method required for this system review |
| **Chatham1997** | Through range computer generated inspiratory muscle training in cystic fibrosis | Mention adults but not age |
| **NCT03190031** | Respiratory Muscle Training in CF Patients | Age mismatch |
| **NCT03737630** | Inspiratory Muscle Training in Individuals With Cystic Fibrosis | Age mismatch;  Study completed 31/1/2020 but no results published. The latest update time is 16/04/2020. Attempted to contact authors but limited contact details available. |
| **NCT04170114** | Comparison of the Efficacy of Comprehensive Respiratory Physiotherapy in Children With Cystic Fibrosis and  Non-Cystic Fibrosis Bronchiectasis | Intervention mismatch，no RMT |
| **NCT05655637** | Effects of Breathing Exercises Combined With Endurance and Strength Training in Patients With Cystic Fibrosis. | Age mismatch  Intervention mismatch，no RMT |

| **NTR2092** | Inspiratory muscle training prior to peripheral muscle training in children and adolescents with Cystic Fibrosis | Study completed 01/09/2013 but no results published. The latest update time is 03/04/2023 Attempted to contact authors but limited contact details available. |
| --- | --- | --- |

Ongoing study
